# Catatonic stupor in 32 years old man diagnosed with schizotypal disorder

**DOI:** 10.1192/j.eurpsy.2024.1524

**Published:** 2024-08-27

**Authors:** I. Ljutica, Z. B. Otasevic, D. Otasevic

**Affiliations:** ^1^Psychiatric Clinic, Clinical Center of Montenegro; ^2^Dusa, Filipovic policlinic; ^3^Dusa, Dusa, Podgorica, Montenegro

## Abstract

**Introduction:**

Stupor is a state of numbness of almost all personality functions, accompanied by stiffness, lethargy and abulia (lethargy). A person in a state of stupor is recognized by the fact that he is constantly silent, does not respond to stimuli at all, refuses food, has a motionless body posture, a face immobile like a mask, a gloomy and absent look. We can call a person who is in a stupor only by calling loudly, shaking hard and similar charms. Catatonic stupor is a state of complete loss of spontaneous and active movement, the patient stands stiffly for hours, sits, does not take food, does not speak but registers everything that is happening around him because his consciousness is not clouded.

**Objectives:**

Here, we report on the case of a 32 year-old man. He was brought in the Emergency Center by his mother with the eyes shut and unresponsive to all sorts of verbal and gestural attempts to elicit any kind of response, with extreme complete body rigidity. He was sweating.

Over several weaks, he developed gradually social withdrowal, motoric stereotypies, loss of apetite, body stiffness. Three days before he was admited to the hospital he stopped eating, drinking water, he was developed body rigidity.

**Methods:**

Case report

**Results:**

He was admitted to a Psychiatric Clinic and first days he was treated with 7,5 mg of lorazepam daily, kariprazin tbl. a 3mg in the morning and olanzapine 10 mg in the evening. Over several days symptoms has diminished.

**Conclusions:**

The patient was reacted very well on the therapy and after several days syptoms diminished. After a month he was released from the hospital. He is in good remission for over a year. He comes regularly for outpatient check-ups

**Disclosure of Interest:**

None Declared

